# Physical Activity and Influenza-Coded Outpatient Visits, a Population-Based Cohort Study

**DOI:** 10.1371/journal.pone.0039518

**Published:** 2012-06-21

**Authors:** Eric Siu, Michael A. Campitelli, Jeffrey C. Kwong

**Affiliations:** 1 Institute for Clinical Evaluative Sciences, Toronto, Ontario, Canada; 2 Department of Family and Community Medicine, University of Toronto, Toronto, Ontario, Canada; 3 Dalla Lana School of Public Health, University of Toronto, Toronto, Ontario, Canada; 4 Public Health Ontario, Toronto, Ontario, Canada; University of Hong Kong, Hong Kong

## Abstract

**Background:**

Although the benefits of physical activity in preventing chronic medical conditions are well established, its impacts on infectious diseases, and seasonal influenza in particular, are less clearly defined. We examined the association between physical activity and influenza-coded outpatient visits, as a proxy for influenza infection.

**Methodology/Principal Findings:**

We conducted a cohort study of Ontario respondents to Statistics Canada’s population health surveys over 12 influenza seasons. We assessed physical activity levels through survey responses, and influenza-coded physician office and emergency department visits through physician billing claims. We used logistic regression to estimate the risk of influenza-coded outpatient visits during influenza seasons. The cohort comprised 114,364 survey respondents who contributed 357,466 person-influenza seasons of observation. Compared to inactive individuals, moderately active (OR 0.83; 95% CI 0.74–0.94) and active (OR 0.87; 95% CI 0.77–0.98) individuals were less likely to experience an influenza-coded visit. Stratifying by age, the protective effect of physical activity remained significant for individuals <65 years (active OR 0.86; 95% CI 0.75–0.98, moderately active: OR 0.85; 95% CI 0.74–0.97) but not for individuals ≥65 years. The main limitations of this study were the use of influenza-coded outpatient visits rather than laboratory-confirmed influenza as the outcome measure, the reliance on self-report for assessing physical activity and various covariates, and the observational study design.

**Conclusion/Significance:**

Moderate to high amounts of physical activity may be associated with reduced risk of influenza for individuals <65 years. Future research should use laboratory-confirmed influenza outcomes to confirm the association between physical activity and influenza.

## Introduction

The protective effects of physical activity against chronic diseases such as coronary artery disease, hypertension, non-insulin-dependent diabetes mellitus, osteoporosis, colon cancer, anxiety, and depression are well established [1]. However, little is known about the potentially protective effects of physical activity on infections, particularly seasonal influenza, which continues to cause substantial morbidity and mortality [2], [3].

Previous studies have mainly examined the relationship between physical activity and upper respiratory tract infections (URTIs), and have focused on small samples of athletes rather than the general population [4]. Among athletes, the association has been described as a J-shaped curve [5], [6]; strenuous exercise such as marathon running can increase susceptibility to URTIs [7], [8], while moderate physical activity can reduce the risk [9]–[12]. Moderate exercise increases immune cell counts and function mildly, whereas strenuous exercise suppresses the immune system about 3–72 hours post-exercise [13]–[18]. In population-based studies of physical activity and URTIs, moderate to high levels of physical activity have been associated with about a 20% lower risk of self-reported URTIs [12], [19].

In the only study that specifically evaluated physical activity and influenza, low to moderate exercise frequency was associated with reduced influenza-associated mortality [9]. However, it is unclear whether more physically active individuals are less likely to be infected with influenza or are less likely to develop complications after influenza infection. We sought to examine the association between physical activity and influenza-coded outpatient visits, as a proxy for influenza infection.

## Methods

### Population, Setting, and Study Design

We conducted a cohort study of Ontario respondents to Statistics Canada’s population health surveys. We included data from the 1996/97 cycle of the National Population Health Survey (NPHS) and cycles 1.1 (2000–01), 2.1 (2003), and 3.1 (2005) of the Canadian Community Health Survey (CCHS) [20], [21]. These cross-sectional surveys collected information on health status, health care use, and health determinants, but excluded persons <12 years of age, institutionalized residents, full-time members of the Canadian Forces, and residents of reserves and certain remote regions [22]. Individuals were selected using a multistage cluster sampling design through telephone and in-person interviews (response rate  = 75–82%) [20]. We excluded survey responders who refused to be linked to health administrative datasets (84% agreed to linkage). We observed negligible differences in terms of age, sex, self-perceived health, and prevalence of self-reported chronic conditions between those who did and did not agree to linkage (data not shown). Encrypted health card numbers were used as unique identifiers to link across datasets.

To assemble our cohort, we included over 12 seasonal influenza epidemics (1996–97 to 2007–08). Individuals were included at the start of each influenza season. Eligible respondents who had completed a survey within three years prior to the start of an influenza season were included for that particular season. As such, an individual could be included in the cohort up to three times. A small group of respondents (790 out of 121,779 [0.6%]) completed more than one survey. For these individuals, we kept their earliest three appearances in the cohort for analysis. All subjects had universal access to physician and hospital services throughout the study period, as well as influenza vaccines starting in fall 2000.

### Ethics Statement

Ethics approval was obtained from the Research Ethics Board of Sunnybrook Health Sciences Centre, Toronto, Canada.

### Data Sources and Definitions

#### Influenza seasons

Laboratory test results (mainly viral culture and direct antigen detection) for influenza that were conducted by sentinel laboratories and reported to the Public Health Agency of Canada were used to define influenza seasons. The beginning and end of each season was defined as the first and last occurrences of two consecutive weeks with at least 5% of specimens testing positive.

#### Physical activity

Physical activity was measured in the NPHS and CCHS using a modified version of the Physical Activity Monitor (PAM), which is based on the Minnesota Leisure-Time Physical Activity Questionnaire (MLTPAQ) [23]. The PAM exhibited very good test-retest reliability (Spearman correlation coefficient (P) = 0.90) [23], comparable to that of the MLTPAQ (0.88–0.92) [24], [25]. Criterion validity of the PAM, using measured maximum oxygen uptake as the criterion, was more modest, but was reasonable when compared to the MLTPAQ (P = 0.36 and 0.47, respectively) [23], [25]. These instruments have been previously used to examine associations between physical activity and health outcomes [26]–[29]. Briefly, respondents were asked if they participated in any of 21 activities over the past three months, and the frequency and duration of each activity [23]. Energy expenditure (EE) was calculated based on the frequency and duration of physical activity and the value of metabolic energy cost, expressed as a multiple of the resting metabolic rate (MET) [30]. The EE values were calculated as EE (kcal/kg/day)  =  ∑[*N_i_D_i_*(METs)/365], where *N_i_* represents the frequency with which the respondents engaged in an activity over a 12-month period, *D_i_* represents the average duration in hours of the activity, and METs are the energy cost of the activity expressed as kilocalories expended per kilogram of body weight per hour of activity [30]. MET values for various activities were specified using the approach adopted from the Canadian Fitness and Lifestyle Research Institute [30]. Individual physical activity levels were classified into three categories based on total average daily energy expenditure: inactive if <1.5 METs/day, moderately active if 1.5–2.9 METs/day, and active if ≥3.0 METs/day [23].

#### Main outcome measure

As a proxy for influenza infection, the primary outcome was physician office or emergency department visits with a diagnosis of influenza (Ontario Health Insurance Plan [OHIP] code 487) during influenza season. These visits were ascertained from the OHIP physician billing claims database, which contains claims from approximately 98% of Ontario physicians [31]. We considered only one event per influenza season per cohort member. To assess the validity of influenza-coded outpatient visits, we obtained and linked data on laboratory specimens tested for influenza between September 1, 2010 and July 31, 2011 to our outpatient dataset. Using polymerase chain reaction (PCR)-confirmed influenza infection as the criterion standard, influenza-coded outpatient visits have a positive predictive value of 71% and a specificity of 99% (unpublished observations). In other settings, influenza-coded ambulatory visits are strongly correlated with influenza virus circulation in the community [32], and a validation study that matched specimen testing data and patient records demonstrated that approximately 50% of outpatient visits coded as influenza were laboratory-confirmed to be positive for influenza A or B [33].

#### Covariates

We obtained the following data from the NPHS and CCHS: smoking status, number of individuals residing in the respondent’s household, marital status, self-reported health, highest level of education, self-reported body mass index, and whether respondents needed help from others with activities of daily living (ADL), including help preparing meals, getting to and from appointments, moving around the house, chores, and personal hygiene. Physical activity at work was assessed by asking respondents to select one of four statements to describe their typical day: “usually sit during the day and don’t walk around very much," “stand or walk quite a lot during the day but don’t have to carry or lift things very often," “usually lift or carry light loads, or have to climb stairs or hills often," or “do heavy work or carry very heavy loads." The calendar season in which the individual responded to the health surveys was also included as a covariate, since leisure-time physical activity varies by season [22].

Ontario’s Registered Persons Database, which contains demographic information about individuals with a valid Ontario health card, was used to determine age, sex, neighborhood income quintile, and rural residence (living in a community with <10,000 inhabitants) [34]. To calculate neighborhood income quintile, respondent postal code was used to determine the average annual income in the surrounding area through linkage with census data [35].

Information regarding previous healthcare utilization (number of outpatient visits in the previous year and number of hospitalizations in the previous three years) was gathered from the OHIP physician billing claims database and the Canadian Institute of Health Information Discharge Abstract Database (CIHI-DAD). CIHI-DAD contains information on diagnoses and procedures for admissions to all acute-care hospitals [36].

Based on an adaptation of the adjusted clinical group classification, comorbidities for patients were assessed if there was evidence of a diagnosis in the outpatient or hospitalization datasets during the previous three years [37]. We included comorbidities that increase the risk of influenza complications, including heart diseases, respiratory diseases, diabetes, cancers, immunodeficiency due to underlying disease and/or therapy, renal diseases, anemia, and lipid disorders [38].

Influenza vaccination status was assessed using the OHIP database. Individuals who received vaccination after the start of an influenza season were considered unvaccinated, however, typically only 5% of vaccines are given after the onset of influenza season.

We assessed influenza vaccination status and demographic, comorbidity, and health care utilization information at the start of each influenza season. Covariates measured using survey responses remained fixed throughout the study.

### Statistical Analyses

Logistic regression, both unadjusted and adjusted for baseline covariates, was used to estimate the risk of influenza-coded outpatient visits during influenza seasons among three physical activity categories, with inactive individuals as the referent. The risk was estimated for all individuals in our cohort, then stratified by those <65 and those ≥65 years.

Several sensitivity analyses were conducted to test the robustness of our findings. We estimated the risk for the active and moderately active groups combined. Since individuals could be included multiple times in the cohort, we used generalized estimating equation (GEE) models to adjust for the correlated nature of the data. Also, since resting energy expenditure declines in the elderly [39], we performed an analysis in which we reduced the threshold for inactivity from <1.5 METs/day to <1.0 METs/day. To assess the specificity of the association of physical activity and influenza, we examined the association between physical activity and dermatitis-coded outpatient visits (OHIP code 691) and periodic health examinations (OHIP code 917) during influenza season. Additionally, to assess an outcome that is less specific for influenza, we examined the association between physical activity and pneumonia or influenza (OHIP code 486 or 487).

Statistical analyses were conducted using SAS 9.1 (SAS Institute INC., Cary, NC). All tests were two-tailed and we used p<0.05 as the level of statistical significance.

## Results

The cohort comprised 121,779 survey respondents who contributed 357,466 person-influenza seasons of observation. Most (94.4%) subjects were included in the cohort three times, while 4.8% twice, and 0.8% only once. Approximately 47.7% of the cohort was categorized as inactive, 24.2% as moderately active, and 25.5% as active (Table 1). Active individuals were younger and more likely to be male, had higher socioeconomic status, had fewer outpatient and hospital visits, had a greater number of individuals in their household, were more likely to have been surveyed in the summer, and were less likely to be married or to be overweight or obese. Active individuals also reported higher levels of activity at work and during the day, and had fewer risk factors for influenza complications, particularly chronic cardiovascular and respiratory diseases, diabetes, cancer, and blood disorders. Fewer of these individuals received influenza vaccines in physician offices. These individuals were less likely to need help with ADLs, and more likely to rate their health favorably compared to the less active groups.

**Table 1:** Baseline characteristics by physical activity level.

	Physical Activity Level		
	Active	Moderately active	Inactive
	≥3.0 METs/day	1.5–2.9 METs/day	<1.5 METs/day
Characteristic^a^	N = 91,155	N = 86,588	N = 170,582
Age (years), mean (SD)	41.1 (19.8)	46.8 (19.0)	50.3 (19.3)
Sex (male)	48,136 (52.8%)	39,038 (45.1%)	71,701 (42.0%)
Rural residence			
No	70,585 (77.4%)	67,438 (77.9%)	131,910 (77.3%)
Yes	20,001 (21.9%)	18,596 (21.5%)	37,667 (22.1%)
Income quintile			
1 (lowest)	15,891 (17.4%)	15,129 (17.5%)	36,289 (21.3%)
2	17,321 (19.0%)	16,815 (19.4%)	35,671 (20.9%)
3	18,457 (20.2%)	17,677 (20.4%)	34,487 (20.2%)
4	18,762 (20.6%)	17,980 (20.8%)	32,810 (19.2%)
5 (highest)	19,912 (21.8%)	18,249 (21.1%)	29,947 (17.6%)
University education			
No	51,650 (56.7%)	44,922 (51.9%)	99,110 (58.1%)
Yes	38,941 (42.7%)	41,153 (47.5%)	70,151 (41.1%)
Self-reported health			
Excellent	27,536 (30.2%)	20,465 (23.6%)	29,329 (17.2%)
Very good	36,812 (40.4%)	34,807 (40.2%)	58,725 (34.4%)
Good/fair/poor	26,789 (29.4%)	31,307 (36.2%)	82,444 (48.3%)
Married	37,106 (40.7%)	43,174 (49.9%)	85,631 (50.2%)
Current smoker	19,614 (21.5%)	19,527 (22.6%)	46,754 (27.4%)
Body mass index			
<18.5	5,097 (5.6%)	3,275 (3.8%)	6,459 (3.8%)
18.5–25	48,142 (52.8%)	40,914 (47.3%)	71,744 (42.1%)
25–30	27,109 (29.7%)	28,615 (33.0%)	55,681 (32.6%)
>30	9,182 (10.1%)	12,230 (14.1%)	32,313 (18.9%)
Work physical activity			
Sit or little walking	14,293 (15.7%)	17,972 (20.8%)	50,514 (29.6%)
Stand or walk lots with no lifting	41,731 (45.8%)	41,198 (47.6%)	74,879 (43.9%)
Lift light loads or climb stair/hills often	27,453 (30.1%)	22,058 (25.5%)	34,187 (20.0%)
Heavy work or lift heavy loads	7,425 (8.1%)	5,150 (5.9%)	10,487 (6.1%)
Requiring help with Activities of Daily Living	6,810 (7.5%)	10,062 (11.6%)	35,151 (20.6%)
Number of household Individuals, mean (SD)	2.9 (1.4)	2.7 (1.4)	2.6 (1.4)
Number of hospitalizations (past three years), mean (SD)	0.2 (0.6)	0.2 (0.7)	0.3 (0.9)
Number of outpatient visits (past year), mean (SD)	7.0 (9.1)	8.2 (10.2)	10.00 (12.5)
Influenza vaccination	14,451 (15.9%)	16,814 (19.4%)	37,396 (21.9%)
Chronic medical conditions			
Cancers	6,089 (6.7%)	7,211 (8.3%)	16,041 (9.4%)
Chronic cardiovascular diseases	19,579 (21.5%)	24,221 (28.0%)	57,166 (33.5%)
Chronic respiratory diseases	22,645 (24.8%)	21,868 (25.3%)	49,669 (29.1%)
Diabetes	4,681 (5.1%)	5,624 (6.5%)	16,453 (9.6%)
Lipid disorder	9,207 (10.1%)	10,474 (12.1%)	21,321 (12.5%)
Immunodeficiency (due to disease and/or therapy)	354 (0.4%)	351 (0.4%)	810 (0.5%)
Anemia	3,285 (3.6%)	3,993 (4.6%)	10,517 (6.2%)
Other disorders	3,980 (4.4%)	5,003 (5.8%)	14,449 (8.5%)
Season of survey completion			
Fall	22,272 (24.4%)	20,065 (23.2%)	37,613 (22.0%)
Spring	23,599 (25.9%)	24,897 (28.8%)	51,747 (30.3%)
Summer	32,072 (35.2%)	25,386 (29.3%)	37,958 (22.3%)
Winter	13,212 (14.5%)	16,240 (18.8%)	43,264 (25.4%)

MET  =  resting metabolic rate.

a – Data are presented as Number (%) of patients unless otherwise specified.

In the entire cohort, 1,706 influenza-coded outpatient visits occurred during influenza seasons. There were 881, 373, and 409 events among inactive, moderately active, and active individuals, respectively (Table 2). Of these events, 1,384 occurred in those <65 years and 322 events in those ≥65 years of age. Increased physical activity was associated with reduced odds of influenza-coded outpatient visits during influenza season. In the adjusted analyses, individuals who were moderately active (OR 0.83; 95% CI 0.74–0.94) and active (OR 0.87; 95% CI 0.77–0.98) had significantly lower odds of an influenza-coded visit. Stratifying by age, the protective effect of physical activity remained significant for individuals <65 years (active: OR 0.86; 95% CI 0.75–0.98, moderately active: OR 0.85; 95% CI 0.74–0.97) but not for individuals ≥65 years.

**Table 2:** Odds ratios and 95% confidence intervals for associations between physical activity level and influenza-coded outpatient visits (adjusted and unadjusted analyses).

		Odds ratio (95% confidence interval)	
Age and physical activity level	Events	Unadjusted	Adjusted^a^
*All ages*			
Inactive	881	1.00	1.00
Moderately active	373	0.83 (0.74–0.94)	0.87 (0.76–0.98)
Active	409	0.87 (0.77–0.98)	0.86 (0.76–0.97)
*<65 years*			
Inactive	688	1.00	1.00
Moderately active	304	0.82 (0.72–0.94)	0.85 (0.74–0.97)
Active	365	0.88 (0.77–0.99)	0.86 (0.75–0.98)
*≥65 years*			
Inactive	193	1.00	1.00
Moderately active	69	0.86 (0.65–1.13)	0.98 (0.73–1.31)
Active	44	0.69 (0.50–0.95)	0.85 (0.60–1.21)

a – All models adjusted for the variables listed in Table 1.

Collapsing the active and moderately active groups together yielded similar results for individuals <65 years (OR 0.85; 95% CI 0.76–0.95) and for those ≥65 years (OR 0.93; 95% CI 0.72–1.2). GEE models to account for repeated observations from the same individuals produced estimates that were nearly identical to those in our original analysis (data not shown). Reducing the threshold of inactivity to <1.0 METs/day for those ≥65 years had no effect on the results (data not shown).

Further analyses revealed no significant association between physical activity and dermatitis-coded outpatient visits or periodic health examinations (Table 3). Stratifying by age, a significant association (OR 1.14; 95% CI 1.02–1.27) was noted for moderately active individuals ≥65 years receiving periodic health examinations. For the less specific outcome of outpatient visits for pneumonia or influenza, a statistically significant reduction was observed for moderately active individuals (OR 0.92; 95% CI 0.85–0.99) but not for active individuals (OR 0.94; 95% CI 0.87–1.02).

**Table 3:** Sensitivity analyses.

	Dermatitis visits		Periodic health examinations		Pneumonia or influenza visits	
	Adjusted^a^ OR		Adjusted^a^ OR		Adjusted^a^ OR	
Age and physical activity level	Events	(95% CI)	Events	(95% CI)	Events	(95% CI)
*All ages*						
Inactive	2321	1.00	5068	1.00	2461	1.00
Moderately active	1122	0.98 (0.91–1.06)	2788	1.01 (0.96–1.06)	934	0.92 (0.85–0.99)
Active	1160	0.99 (0.92–1.06)	2587	1.04 (0.99–1.09)	918	0.94 (0.87–1.02)
*<65 years*						
Inactive	1652	1.00	3931	1.00	1391	1.00
Moderately active	881	1.02 (0.94–1.11)	2160	0.96 (0.91–1.02)	614	0.90 (0.82–0.99)
Active	987	1.02 (0.94–1.11)	2028	1.01 (0.96–1.07)	691	0.93 (0.84–1.03)
*≥65 years*						
Inactive	669	1.00	1137	1.00	1070	1.00
Moderately active	241	0.86 (0.72–1.04)	628	1.14 (1.02–1.27)	320	0.96 (0.84–1.10)
Active	173	0.92 (0.79–1.07)	559	1.07 (0.97–1.19)	227	0.95 (0.81–1.11)

OR  =  Odds Ratio; CI  =  Confidence Interval.

a – All models adjusted for the variables listed in Table 1.

## Discussion

During periods of seasonal influenza activity, we found moderately active (1.5–2.9 METs/day) and active (≥3.0 METs/day) individuals to be approximately 15% less likely to have an influenza-coded physician office or emergency department visit compared to inactive individuals. When stratified by age, we observed similar findings among individuals <65 years but not ≥65 years. The various sensitivity analyses conducted demonstrated the robustness of our findings. Among individuals <65 years, moderately active and active individuals were not more likely than inactive individuals to visit physicians for non-influenza-related conditions such as dermatitis or periodic health examinations during influenza season, suggesting that the observed protective effects of physical activity against influenza-coded outpatient visits are not from underlying differences in health status or healthcare seeking behaviour. However, moderately active individuals ≥65 years were more likely to have periodic health examinations during the influenza season, which suggests the presence of “healthy user" bias for this outcome within this age group. This was confirmed in a *post hoc* analysis indicating that both moderately active and active individuals were more likely to have periodic health examinations during non-influenza periods.

The protective effects of physical activity among younger individuals but not older adults may be explained by age-related changes in immune function. Aging is linked to declines in the ability to defend against pathogens [40], and has been associated with increased morbidity and mortality from infectious diseases in the elderly [40]–[41]. Additionally, age-related declines in immune response to influenza vaccines are well documented [42]–[44]. The reduced immune function of the elderly may prevent them from receiving any immune system benefits from physical activity.

To our knowledge, this is the first epidemiologic study that has examined the relationship between physical activity and influenza-related morbidity during seasonal influenza epidemics. Previous studies have mostly focused on upper respiratory tract infections (URTIs) with an emphasis on athletes [4], and only a few focused on the general population [12], [19], [45]. Our finding of a 15% reduction in influenza-coded outpatient visits is similar to the 20% reduction in URTIs observed in population-based studies, although those studies used self-reported outcome measures [12], [19], [45]. Only one other study assessed the association between physical activity and influenza, and the outcome was influenza-associated mortality [9]. Although a beneficial effect was found, our study suggests a protective effect at a much earlier stage than mortality.

This study had several limitations. First, our outcome measure was influenza-coded outpatient visits rather than laboratory-confirmed influenza infections, which would be the most ideal outcome measure but is challenging to incorporate in population-based studies because influenza infection is infrequently confirmed. Although in another study only 50% of outpatient visits coded as influenza were actually laboratory-confirmed to be positive for influenza [33], we found that in Ontario these visits have very high specificity and reasonably high positive predictive value for PCR-confirmed influenza infection. Moreover, since this misclassification is likely to have occurred equally across the physical activity groups, our estimates would be biased towards the null, and therefore the results of this study most likely underestimate the true protective effect of physical activity against influenza infection. A second limitation is that measurement of physical activity and certain covariates relied on self-report, and verification of subject responses was not possible. However, prior studies have demonstrated the validity of these measures [23], [46]–[48]. Also related to the survey data, these measures may have changed over the study period and could not be updated at the start of each influenza season for included participants. However, our decision to restrict the analyses to individuals who had completed a survey within three years prior to the start of an influenza season was guided by literature that suggests physical activity levels remain stable over three years [49]. A fourth limitation is that this study focused mainly on leisure time physical activity. Data on work-related physical activity was limited as both the NPHS and CCHS had only one question that addressed physical activity at work. A fifth limitation is that we were underpowered to more finely stratify our age categories in order to determine which specific age groups (e.g., teenagers, young adults, middle-aged individuals) contributed most to our finding of reduced influenza-coded outpatient visits in those <65 years. Additionally, although the MET value is ubiquitous throughout the physical activity literature, its use across age groups, particularly in the elderly, has not been properly validated [39], [50]–[51]. Finally, as with all observational studies, the observed associations might be attributed to residual confounding.

Linking population-based physical activity data to health administrative databases was one major strength of our study. Doing so allowed us to determine the effect of physical activity on influenza at the population level using a more objective outcome measure (physician visit for influenza) rather than patient self-report of acute respiratory symptoms. Additionally, we included multiple influenza seasons in our study, which is important since the severity of influenza season varies between years.

Although the benefits of physical activity in preventing chronic conditions are well established, its impacts on infectious diseases have been less clearly defined. The results of this study suggest that moderate to high amounts of physical activity may be associated with reduced risk of influenza for individuals <65 years. Future research should ideally use laboratory-confirmed influenza outcomes to confirm the association between physical activity and influenza infection. Public health authorities and clinicians should work toward a common goal of increasing physical activity and the public’s awareness of its benefits. These actions may help to mitigate the health and economic burden caused by influenza.
